# Dielectric Tunability Properties in (110)-Oriented Epitaxial 0.5Ba(Ti_0.8_Zr_0.2_)O_3_-0.5(Ba_0.7_Ca_0.3_)TiO_3_ Thin Films Prepared by PLD Method

**DOI:** 10.3390/ma13214771

**Published:** 2020-10-26

**Authors:** Bing Luo, Yiwen Xu, Fuzeng Zhang, Tingting Wang, Yingbang Yao

**Affiliations:** 1 China Southern Power Grid, Guangzhou 510623, China; luobing@csg.cn (B.L.); zhangfz@csg.cn (F.Z.); wangtingting@csg.cn (T.W.); 2 School of Materials and Energy, Guangdong University of Technology, Guangzhou 510006, China; xuyw1204@163.com

**Keywords:** BZT-BCT thin films, pulsed laser deposition, ferroelectrics, dielectric tenability

## Abstract

Epitaxial 0.5Ba(Ti_0.8_Zr_0.2_)O_3_-0.5(Ba_0.7_Ca_0.3_)TiO_3_ (BZT-BCT) thin films with single-crystal perovskite structure have been grown by pulsed laser deposition (PLD) on the (110) SrRuO_3_/SrTiO_3_ substrates. Temperature-dependent dielectric measurements show obvious characteristics of a diffused phase transition. Typical *P*-*E* hysteresis loops with a distinct ferroelectric imprint phenomenon are observed in these BZT-BCT thin films with a remnant polarization of 2.0 μC/cm^2^ and coercive field of 187 kV/cm. Small leakage currents (<1 × 10^−6^ A/cm^2^) are obtained in these thin films under an electrical field of 240 MV/m. These BZT-BCT thin films have shown large dielectric tunability values ranging from 75.8% to 85.7%, under a wide temperature range from 200 K to 330 K and a frequency range between 100 Hz and 100 kHz, which shows their good temperature and frequency stability. Such excellent dielectric tunability properties in these (110)-oriented BZT-BCT thin films promise their great potentials in practical phase shifter applications.

## 1. Introduction

As is well-known, lead zirconate titanate (PZT) materials exhibit excellent piezoelectric and ferroelectric properties and have been widely used in microelectronic sensors or micro-electro-mechanical system (MEMS) [1,2,3]. However, the poisonous lead oxide used for preparing PZT materials can cause serious environmental contamination, which restricts the further development of PZT materials. Therefore, many efforts have been devoted to the development of lead-free ferroelectrics. Among them, BaTiO_3_-based lead-free ferroelectric materials arouse intense research interests, and these materials exhibit the promising potential to replace the PZT ceramics [4,5,6]. Series of BaTiO_3_-based ceramics, such as Ba(Ti_1−*x*_Zr*_x_*)O_3_ (BZT) or Ba*_x_*Sr_1−*x*_TiO_3_ (BST), have been developed through the suitable addition or substitution of Ba and Ti with different elements, including Zr or Sr [7,8,9]. Ren et al. developed (1−*x*) Ba(Ti_0.8_Zr_0.2_)O_3_-*x*(Ba_0.7_Ca_0.3_)TiO_3_ (BZT-BCT) ceramic family and found that a morphotropic phase boundary (MPB) existed at *x* = 0.5 where a large piezoelectric response (*d_33_* ~ 620 pC/N) was obtained. Their *d_33_* values are comparable to those of the PZT ceramics [1,2,3,4,5,6]. This kind of material is one of the most promising candidates for lead-free piezoelectric ceramics with the apparent drawback of its low Curie temperature (below 373 K).

Most of the research works have been focused on these lead-free bulk materials regarding the preparation methods, structural and piezoelectric or ferroelectric performances, etc. [10,11,12]. Sol-gel technique [8] and solid-sate reaction method [11,12] are usually used to prepare ceramics of BZT, BST, or BZT-BCT systems. These bulk lead-free ceramics present a perovskite ABO_3_ structure with different atomic site occupation and show enhanced piezoelectric or ferroelectric performances. On the other hand, thin-film ceramic materials are also widely used to fabricate various ferroelectric devices like microwave phase shifters or micro-piezoelectric motors. It is necessary to choose an appropriate method to prepare high-quality thin films of lead-free ceramics. Many works have been reported to fabricate the polycrystalline BZT or BZT thin films by sol-gel [6,13], screen printing [7,14], or sputtering method [15]. For BZT-BCT materials, there are also several reports on thin film preparation and characterization [16,17,18,19,20]. Luo et al. prepared (001)-, (110)-, and (111)-oriented epitaxial BZT-BCT (*x* = 0.5) thin films on La_0.7_Sr_0.3_MnO_3_ (LSMO)-coated (001), (110), and (111) SrTiO_3_ (STO) single crystal substrates by radio-frequency magnetron sputtering method and studied their piezoelectric properties using piezoelectric force microscopy (PFM) [16]. Kolekar et al. used a pulsed laser deposition (PLD) method to deposit BZT-BCT (*x* = 0.5) polycrystalline thin films on Pt/Ti/SiO_2_/Si substrates and obtained a high remnant polarization of ~37 μC/cm^2^ using their homemade measurement system [17]. Puli et al. prepared (001)-oriented epitaxial BZT-BCT (*x* = 0.15) thin films on La_0.5_Sr_0.5_CoO_3_ (LSCO)-buffed MgO (100) single crystal substrate by PLD and studied their temperature-dependent conduction mechanisms [18]. In another report, they prepared BZT-BCT (*x* = 0.5) polycrystalline thin films on Pt/Ti/SiO_2_/Si substrates and achieved a giant recoverable energy-storage density of 93.52 J/cm^3^ [19]. The room-temperature dielectric tunability is also measured at 10 kHz under an DC electrical field around 100 MV/m, which is ~28% and ~75% for the epitaxial (001)-oriented BZT-BCT (*x* = 0.15) and polycrystalline BZT-BCT (*x* = 0.5) thin films, respectively [18,20]. Lin et al. prepared (001)-oriented epitaxial BZT-BCT (*x* = 0.5) thin films on SrRuO_3_ (SRO)-coated (001) SrTiO_3_ single crystal substrates using the PLD method and studied the oxygen partial pressure during the PLD process on the ferroelectric and piezoelectric properties of their films [20]. A remnant polarization and effective piezoelectric coefficient of 14.5 μC/cm^2^ and *d_33_* = 96 ± 5 pm/V are observed.

Based on the published literature, we found that BZT-BCT materials could exhibit large dielectric tunability at room temperature, depending on their composition [18,19]. However, there is a scarcity in the studies on temperature-dependent dielectric tunability properties, deserving more detailed investigations. In this work, we used the PLD method to prepare epitaxial BZT-BCT (*x* = 0.5) thin films on the (110) single crystal SrTiO_3_ (STO) substrates with bottom electrode SrRuO_3_ (SRO) buffer layer. We chose such a composition due to the fact that it exhibits the best piezoelectric properties in the BZT-BCT material family [4]. The (110) orientation was chosen because it is the naturally strongest peak from the X-ray diffraction pattern of perovskite ferroelectrics. The current research focused on the dielectric tunability properties of these epitaxial BZT-BCT thin films in a wide temperature range, i.e., from 10 K to 360 K, and in a wide frequency range, i.e., from 100 Hz to 100 kHz. Meanwhile, these (110)-oriented BZT-BCT thin films’ crystal structure, microstructure, phase transitions, and ferroelectric properties were also systematically studied.

## 2. Experimental

A BZT-BCT (*x* = 0.5) ceramic target was prepared by a conventional solid-sate-reaction method using reagent-grade BaCO_3_, CaCO_3_, ZrO_2_, and TiO_2_ (from Sigma-Aldrich) as raw materials. The target ceramic composition was 0.5Ba(Ti_0.8_Zr_0.2_)O_3_-0.5(Ba_0.7_Ca_0.3_)TiO_3_. The epitaxial BZT-BCT thin films were deposited on the (110)-oriented single crystal SrTiO_3_ substrates buffered with a conductive SrRuO_3_ layer (as a bottom electrode) by PLD method. The substrate temperature was kept at 1123 K during the PLD process. Under an oxygen pressure of 5 × 10^−2^ torr, the KrF excimer laser (CompexPro 205F, Coherent Inc., Santa Clara, CA, USA) was operated and focused on the desired target at a frequency of 8 Hz and with an energy density of about 2.5 J/cm^2^. These processing parameters were chosen based upon the published reports on BZT-BCT thin film by PLD [17,19]. The number of laser shots was 48,000, corresponding to a thickness of 100 nm. After the deposition was finished, the films were in-situ annealed under an oxygen pressure of 700 torr for 30 min, and then the temperature was decreased from 1123 K to 873 K at a temperature rate of 5 K/min and, finally, cooled to room temperature with a rate of 10 K/min. This in-situ annealing process was expected to reduce the oxygen vacancies as well as the residual stresses, which were developed during the thin film growth.

The crystal structure of the BZT-BCT films was characterized by X-ray diffraction (XRD, Bruker D8 Discover diffractometer, Karlsruhe, Germany) with a Cu Kα radiation source (*λ* = 1.5406 Å). *φ* scan was also performed to reveal the epitaxial nature of the thin films. The surface roughness was analyzed by atomic force microscopy (AFM, Agilent 5500, Santa Clara, CA, USA). Au top electrodes with a diameter of 0.2 mm were deposited on the surface of the BZT-BCT films by e-beam evaporation technique for the measurements of their dielectric and ferroelectric properties. The room-temperature *P*-*E* hysteresis loops, *I*-*V* curves, and capacitance-vs.-voltage (*C*-*V*) curves were measured in a voltage range of 2–24 V by a ferroelectric tester (aixACCT TF Analyzer 2000, Aachen, Germany). The temperature-dependent dielectric tunability properties for the BZT-BCT films were measured by an LCR meter (HIOKI IM 3533-01, Nagano, Japan) and a temperature-controlled chamber (from 10 K to 400 K) in a physical properties measurement system (PPMS, Quantum Design, San Diego, CA, USA), both of which were controlled by a homemade Labview program.

## 3. Results and Discussions

**Figure 1:**
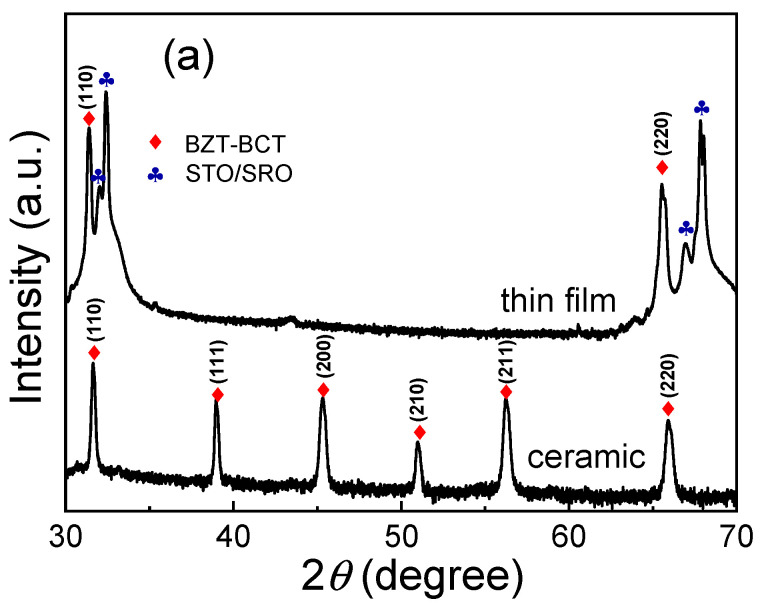

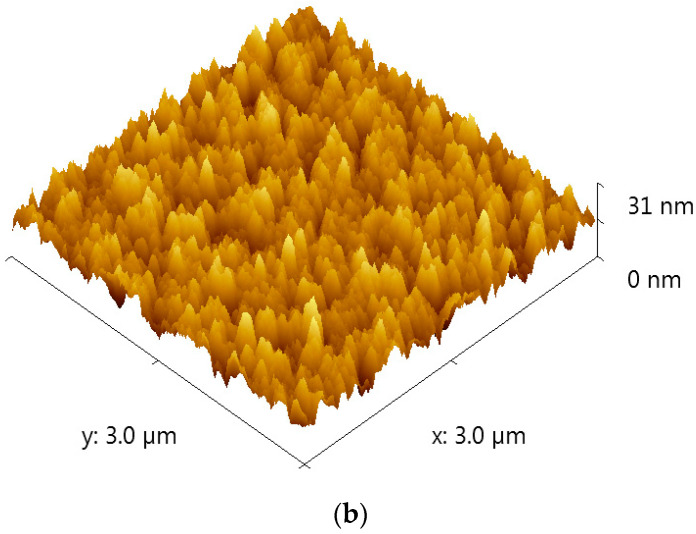
(**a**) XRD patterns of the BZT-BCT ceramic target and the PLD-deposited BZT-BCT thin films; (**b**) surface profile of the BZT-BCT thin film obtained by AFM.

**Figure 2:**
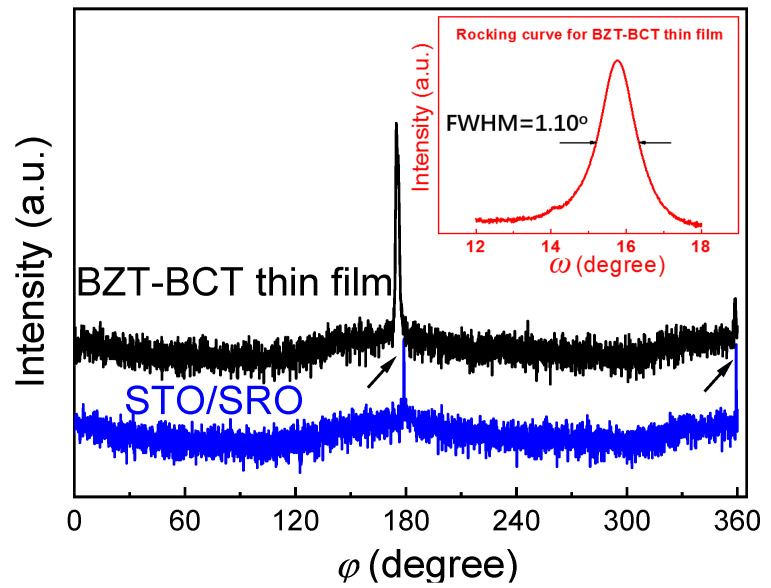
In-plan *φ* scan patterns for the (010) plane of both the substrates and PLD-deposited BZT-BCT thin films (the rocking curve of the BZT-BCT thin film is shown in the inset). The arrows indicate the two peaks in the *φ* scan.

Figure 1a shows the XRD patterns of the BZT-BCT ceramic target and PLD-deposited thin films. As can be observed in Figure 1a, the ceramic target exhibits a pure polycrystalline perovskite structure with all diffraction peaks coincident with the standard patterns [4]. For the thin film sample, besides the diffraction peaks from the (110)-oriented SrRuO_3_-coated SrTiO_3_ substrate, there are other two peaks with 2*θ* values of 31.1° and 65.0°, which are the characteristic peaks of (011) and (022) for the BZT-BCT thin film. These observations indicate the preferred orientation along the (110) direction in the PLD-deposited thin films. Figure 1b shows a typical AFM 3D-view of the surface profile of the BZT-BCT thin film. It can be seen that the surface of the thin film is very clean and dense, free of impurities or cracks. The roughness of the thin film is measured to be *S_a_* = 2.86 nm (root mean square roughness: 3.61 nm). This roughness value is comparable to those epitaxial BZT-BCT thin films prepared by the sputtering or PLD, i.e., from 2–4 nm [16,18]. To further investigate the epitaxy property of the BZT-BCT thin films, in-plan *φ* scan measurements for the (010) plane of both the substrates and thin films are performed [16]. The results are shown in Figure 2. Both of the *φ* scan patterns from the substrate and thin-film show an obvious two-fold symmetry with the 180° interval of two major peaks, indicating a cubic-on-cubic epitaxial relationship between the thin film and the underlying substrate. The inset in Figure 2 presents the rocking curve of the thin film, showing a small full-width at half-maximum (FWHM) value of 1.10°. Such a broad rocking curve indicates a large mosaic spread of BZT-BCT films on the underlying substrate [21]. Indeed, it is reported that the surface roughness of the (110)-oriented and (111)-oriented BZT-BCT thin films is larger than that of the (001)-oriented BZT-BCT thin film, which is believed due to the differences in surface energy (lowest for (001) surface) and the resulting growth behavior [16].

**Figure 3:**
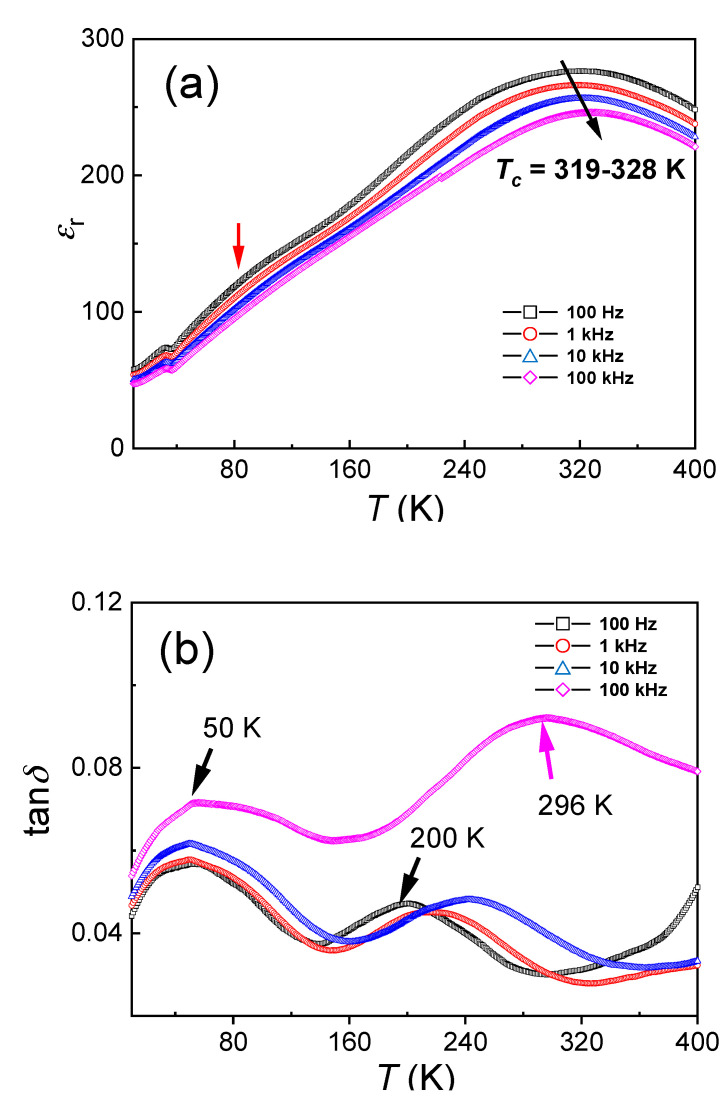
Temperature-dependent (10–400 K) dielectric constant (*ε_r_*) (**a**) and loss tan*δ* (**b**) of the BCT-BZT film at a frequency range from 100 Hz to 100 kHz.

Temperature-dependence of the dielectric constant (*ε_r_*) and loss tan*δ* of the (110)-oriented epitaxial BZT-BCT thin films are measured at a frequency range from 100 Hz to 100 kHz and a temperature range from 10 K to 400 K. The results are shown in Figure 3. It can be found, from Figure 3a, that the dielectric constant is decreased slightly with increasing frequency from 100 Hz to 100 kHz in the whole temperature range, reflecting a slight frequency dispersion of the dielectric property. It can be also observed that the dielectric constant increases obviously with the temperature and reaches a maximum value at 319 K to 328 K, with the measurement frequency from 100 Hz to 100 kHz, respectively. This is due to the ferroelectric (tetragonal) to the paraelectric (cubic) phase transition of the BZT-BCT material [4,20]. In the dielectric loss spectra, this phase transition temperature variation is even much larger, ranging from 200 K (100 Hz) to 296 K (100 kHz), as shown in Figure 3b. These dielectric measurements indicate typical diffused phase transition characteristics, and thus a relaxor-like behavior for these (110)-oriented epitaxial BCT-BZT thin films is confirmed. Puli et al. also investigated the temperature-dependent dielectric constants/losses of BZT-BCT (*x* = 0.5) ceramics as well as polycrystalline thin films (thickness: 360 nm) on Pt-coated Si substrates [20]. In the bulk ceramics sample, they observed three smeared phase transitions at 205 K, 270 K, and 383 K, which were assigned to rhombohedral-orthorhombic, orthorhombic-tetragonal, and tetragonal-cubic phase transitions, respectively. Yet, no obvious phase transitions can be even observed in their thin-film samples. Moreover, in other reports, for the bulk BZT-BCT (*x* = 0.5) ceramics, no orthorhombic phase is clearly observed in their temperature-dependent dielectric curves [4,22,23,24,25], where the rhombohedral-tetragonal and tetragonal-cubic phase transition temperature is found to be 300–310 K and 363–370 K, respectively. Such variations in these transition temperatures should be related to their various processing parameters, for example, the sintering temperatures and dwelling durations, etc. [4,22,23,24,25]. On the other hand, Damjanovic et al. observed another anomaly around 280K through a detailed analysis of the temperature-dependent dielectric and elastic coefficients [26]. Moreover, Keeble et al. also reported a usual structural phase transition at 260 K through a high-resolution synchrotron X-ray powder diffraction study in the BZT-BCT (*x* = 0.5) ceramics [27]. They assigned this phase transition to rhombohedral-orthorhombic, and the other two-phase transitions at 300 K and 366 K were assigned to orthorhombic-tetragonal and tetragonal-cubic, respectively. Based upon these published data, the broad phase transition (at 319–328 K) in our epitaxial BZT-BCT (*x* = 0.5) thin films may be related to locally un-uniform tetragonal-cubic structural transitions or local structural order-disorder transitions [24,25,28,29,30]. It is already known that the ferroelectric phase transition temperatures in thin films are usually lower than those of their bulk counterparts, and the small grain size, lattice defects, and residual stress may account for this [31]. Meanwhile, the frequency-dependence of the dielectric constants in our samples may be related to such local imperfections, which lead to the broad distribution of relaxation times and, thus, the strong frequency dispersion [32,33].

Another dielectric anomaly around 80 K, as indicated by an arrow in Figure 3a, is observed. It is more prominent in the tan*δ*(*T*) curve (the peak around 50 K), as shown in Figure 3b. This phase transition is not previously observed in the BZT-BCT thin films or ceramics [4,16,17,18,19,20,22,23,24,25,26,27]. The reason is that the measurement temperatures for the published dielectric data are limited in the range of 170–470 K [20,22,23,24,25,26]. Synchrotron diffraction studies show that there is no crystal structure change below 260 K in the BZT-BCT (*x* = 0.5) ceramics [27]. However, for the individual component BZT or BCT, there are phase transitions in such temperature range, i.e., 50–100 K [34,35]. Therefore, it is hard to assign this phase transition to a particular origin, and it may be related to one of its individual components. More detailed investigations need to be done in order to clarify this phase transition and its origin. This will be done in the near future and published in another report.

**Figure 4:**
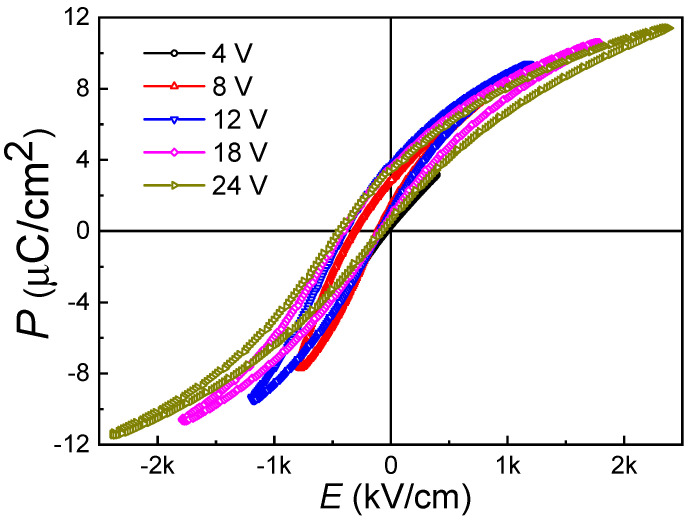
Room-temperature *P*-*E* hysteresis loops of the (110)-oriented epitaxial BZT-BCT thin films.

Figure 4 presents the room-temperature *P*-*E* hysteresis loops of the (110)-oriented epitaxial BZT-BCT thin films with a maximum applied voltage of 24 V (corresponding to an electrical field of 240 MV/m). The frequency of the applied field is fixed at 1 kHz. The shapes of the loops in Figure 4 show characteristics of a slim hysteresis (small remnant polarization and coercive field), with the remnant polarization (*P_r_*) of 2.01 μC/cm^2^, the saturation polarization (*P_s_*) of 11.40 μC/cm^2^, and the coercive field (*E_c_*) of 187 kV/cm. The remnant polarization in our thin film is comparable to those (110)-oriented BZT-BCT (*x* = 0.5) thin films prepared by the sputtering method (~2.39 μC/cm^2^, film thickness: 155 nm, substrate: LSMO/STO) [16]. The coercive field in our sample is similar to that observed in polycrystalline BZT-BCT (*x* = 0.5) thin films on Pt-coated Si substrate (~180 kV/cm, film thickness: 360 nm) [20]. The film thickness and the processing parameters will affect these ferroelectric properties greatly. For example, Lin et al. applied different oxygen pressures (50–350 mTorr) in the PLD process to prepare BZT-BCT (*x* = 0.5) thin films (thickness: 280 nm) and found that remnant polarization could be changed in several folds, i.e., from 5.1 μC/cm^2^ to 14.5 μC/cm^2^ [19].

The other point worthy of notice in Figure 4 is that all the hysteresis loops show a strong imprint behavior, i.e., *P*-*E* loops exhibiting negative shifts along *E*-axis (or positive shift along *P*-axis). At zero applied field, an initial remnant polarization of +0.67 μC/cm^2^ is observed, and there is a −245 kV/cm shift in the coercive field. Such behavior is very stable after many cycles of polarization switching. Usually, an internal field is accounted for this phenomenon. Other reports show that in BZT-BCT, bulk ceramics [4,11,22,23,24,25] or polycrystalline thin films [6,16,17,18,19,20] with a similar composition do not exhibit such imprint behavior in their hysteresis loops. The imprint behavior in ferroelectric ceramics or thin films has been studied experimentally and theoretically [36,37,38,39,40,41]. There are several accepted models, which can be used to explain the imprint phenomena in ferroelectric materials with different forms (bulk or thin-film). For example, the oxygen-vacancy-related defect-dipole model can be used for bulk ceramics or high-quality thin films [36,38]. The electrical field within a thin interface layer (also called interfacial screening model) is proven to be successful in interpreting the enhancement of imprint due to external bias or optical illumination in PZT thin films [38,39]. Theoretical simulations also demonstrate that, in addition to a non-switching interface layer, the stress from the underlying substrate (lattice mismatch effects) can also lead to severe imprint behavior [40]. For thin film samples, it is thus generally accepted that their imprint behavior is mainly related to the non-switching layer between the thin film and the electrodes (bottom and top) [37,38,39,40,41]. This non-switching interface layer is believed to be formed by the relaxation of lattice misfit strain, especially in the epitaxial thin film and of Schottky-barrier nature [37,40]. Although the imprint behavior is detrimental to ferroelectric memory applications, such remnant polarization under zero voltage can generate a strong electrostatic field and may find applications in tuning heterojunctions’ properties without applying an external electrical field when another layer is grown upon the BZT-BCT thin films.

**Figure 5:**
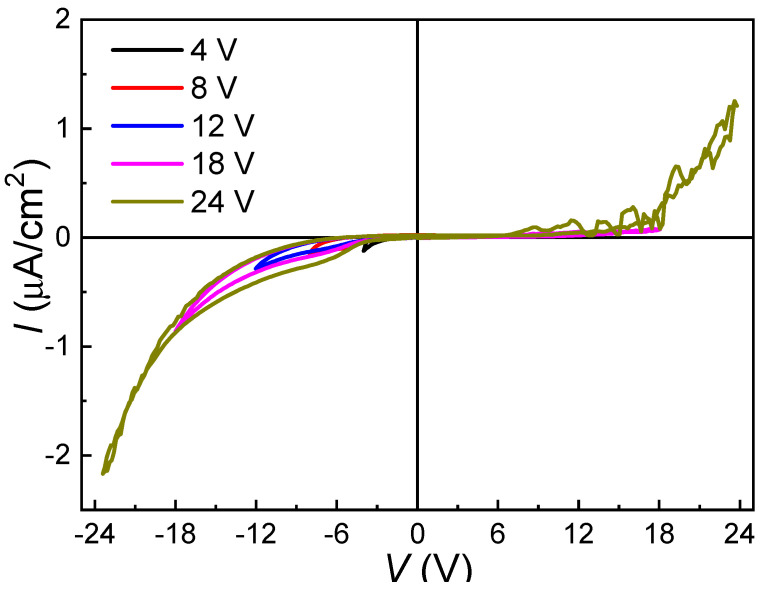
Room-temperature *I*-*V* curves of the (110)-oriented epitaxial BZT-BCT thin films.

Figure 5 shows the room-temperature *I*-*V* curves of the (110)-oriented epitaxial BZT-BCT thin films measured at a voltage range from 4 V to 24 V (corresponding to an electrical field of 40 to 240 MV/m). Very small leakage currents (~2 μA/cm^2^) are observed when the thin films undergo an electrical field as high as 240 MV/m (i.e., 24 V). The leakage current in our sample is much lower than those published results for BZT-BCT thin films, i.e., 6.2 × 10^5^ μA/cm^2^ at 208 MV/m (BZT-BCT, *x* = 0.15, thickness: 360 nm) [18], 5.5 × 10^5^ μA/cm^2^ at 53 MV/m (BZT-BCT, *x* = 0.5, thickness: 280 nm) [19], and 20 μA/cm^2^ at 52 MV/m (BZT-BCT, *x* = 0.5, thickness: 360 nm) [20]. Another feature is the asymmetric behavior of the *I*-*V* curves, which should be related to the different top (Au) and bottom (SrRuO_3_) electrodes. Considering the slim *P*-*E* hysteresis loops and the high dielectric breakdown strength (very stable at 240 MV/m), one can say that these BZT-BCT thin films are very promising in dielectric energy storage applications [20].

**Figure 6:**
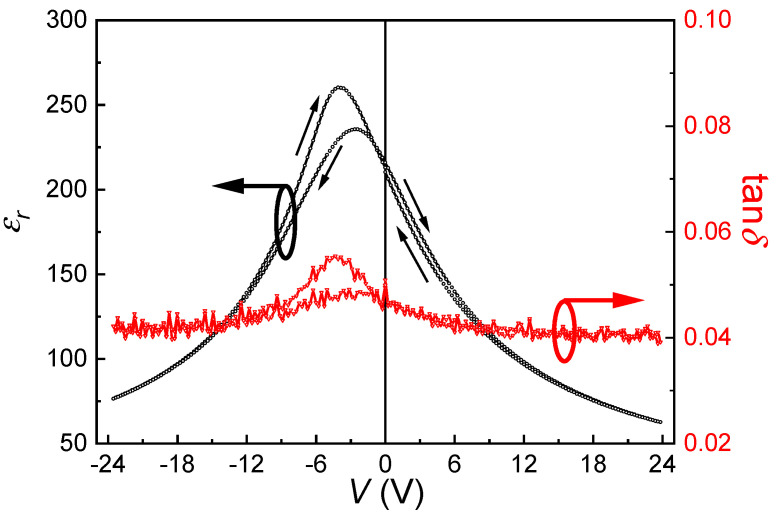
Room-temperature $\varepsilon_{r}$-V curves for the (110)-oriented epitaxial BZT-BCT thin films.

The room-temperature dielectric tunability *ε_r_*(V) curves for the (110)-oriented epitaxial BZT-BCT thin films are shown in Figure 6. The external DC field is swept very slowly (step: 0.24 V, dwelling time at each step: 2 s) from 0 V to +24 V, then +24 V to −24 V, and finally back to 0 V (as indicated by the arrows in the figure). The dielectric constant and loss are measured by a superposed AC signal (500 mV amplitude) at 100 kHz. The dielectric constant is found to be greatly suppressed by the external DC electrical field (e.g., from 260.1 at −3.85 V to 76.5 at −24 V), while the dielectric loss does not change too much (e.g., 0.055 at −3.85 V to 0.042 at −24 V) with the external field. Asymmetric behavior in the field-dependent dielectric properties is clearly observed, which should be related to the ferroelectric imprint in the sample (see Figure 4).

The dielectric tunability is normally defined by the following equation:

(1) $$\mathrm{Tunability}\;=\;\left[\varepsilon_{r}\left(0\right)\;-\;\varepsilon_{r}\left(V\right)\right]/\varepsilon_{r}\left(0\right)$$

where $\varepsilon_{r}$(0) and $\varepsilon_{r}$(V) represent the dielectric constant at zero and an externally applied voltage, respectively. Usually, the $\varepsilon_{r}$(0) is the maximum value in the $\varepsilon_{r}$(V) curve for a conventional bulk ceramic sample. Since in our thin films, there is a severe imprint (or shift of the *P*-*E* loop) problem, we use the dielectric constant maximum as $\varepsilon_{r}$(0) in the above equation. Thus, at room temperature and 1 kHz, the tunability of our thin-film sample is calculated to be 70.6% under an applied voltage of 24 V. Such external field-dependent dielectric property can find applications in electronic phase shifters or filters, where the capacitance of the device needs to be electrically tuned [42,43,44].

**Figure 7:**
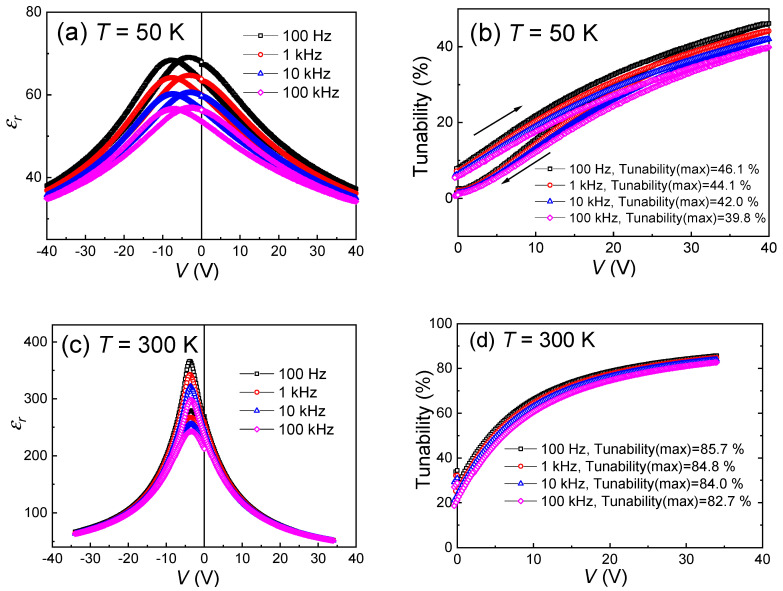

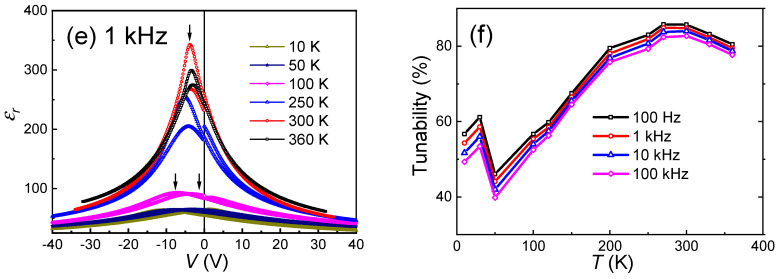
The temperature- and frequency-dependent $\varepsilon_{r}\left(V\right)\;$curves and calculated tunability values: (**a**) the $\varepsilon_{r}\left(V\right)\;$curves at different frequencies measured at 50 K; (**b**) tunability values at 50 K; (**c**) the $\varepsilon_{r}\left(V\right)\;$curves at different frequencies measured at 300 K; (**d**) tunability values at 300 K; (**e**) the temperature-dependent $\varepsilon_{r}\left(V\right)\;$curves measured from 10 K to 360 K at 1 kHz; (**f**) the tunability values as a function of temperature from 10 K to 360 K, measured at a frequency range from 100 Hz to 100 kHz.

In order to further investigate the dielectric tunability performance of the (110) epitaxial BZT-BCT thin film sample, temperature- and frequency-dependent $\varepsilon_{r}\left(V\right)\;$curves in a temperature range from 10 K to 360 K and a frequency range from 100 Hz to 100 kHz are measured. Figure 7a–d show the typical $\varepsilon_{r}\left(V\right)\;$curves and the calculated tunability values (at different frequencies) under the measurement temperature of 50 K (a, b) and 300 K (c, d), respectively. It can be seen with the increasing measurement frequency, the dielectric constant, as well as the tunability, are both decreased. For example, at the measurement temperature of 50 K, the maximum tunability at 40 V (~400 MV/m) is decreased from 46.1% measured at 100 Hz to 39.8% measured at 100 kHz, as shown in Figure 7b. At 300 K, the maximum tunability at 34 V (~340 MV/m) is decreased from 85.7% measured at 100 Hz to 82.7% measured at 100 kHz, as shown in Figure 7d. Regarding the $\varepsilon_{r}\left(V\right)\;$curves measured at different temperatures, one can find that at low temperatures, the two peaks are much more broadened, and the field difference between these two peaks are also increased, i.e., from 0.2 V at 300 K to 3.4 V at 50 K (at the measurement frequency of 100 Hz), as shown in Figure 7a,c. The two peaks correspond to the polarization switching point, i.e., coercive field +*E_c_* and −*E_c_*. The broadening of these peaks implies that the polarization switching slows down at low temperatures. The increase in the separation of the two peaks indicates an increase in the coercive field at low temperatures. Anyway, the polarization switching kinetics determine the dielectric behavior of the sample under DC fields at different temperatures. To further investigate the temperature effects on dielectric properties for these BZT-BCT thin films, we have measured the $\varepsilon_{r}\left(V\right)\;$curves from 10 K to 360 K at 1 kHz, and the results are shown in Figure 7e. It can be found that at the temperature below 100 K, the peaks in the $\varepsilon_{r}\left(V\right)\;$curves are much broader than those measured at higher temperatures. The calculated tunability values at different temperatures, as well as different measurement frequencies, are summarized in Figure 7f. It can be seen that these BZT-BCT thin films have shown large tunability values ranging from 75.8% to 85.7%, measured at a temperature range between 200 K to 330 K and a frequency range between 100 Hz to 100 kHz. These tunability values are comparable to the well-known (Ba*_x_*Sr_1−*x*_)TiO_3_ (~80%) or Ba(Zr*_x_*Ti_1−*x*_)O_3_ (~86%) materials [8,45] and larger than those of the published dielectric tunability values of BZT-BCT (*x* = 0.15) ceramics (~82%) [46] and BZT-BCT (*x* = 0.5) thin films (75%) [20]. The temperature-dependence of the dielectric tunability is related to the amount of switchable polarizations and their switching dynamics at different temperatures [42,43,44]. A larger switchable polarization and a faster switching will lead to a higher tunability value. The polarization switching is usually faster at higher temperatures [47]. Therefore, this will, at least in part, account for the increase of tunability with temperature. However, the drastic drop around 50 K should be related to the unknown phase transitions (as shown in Figure 3), where the polarization value may decrease and leads to the decrease of the tunability. More investigations need to be done in order to clarify the origin of this anomaly. Above the Curie temperature, the tunability value will definitely decrease with temperature because of the diminishing of the polarization. These slight variations in tunability values measured from such a broad temperature and frequency range prove the excellent temperature and frequency stability of the dielectric tunability in our epitaxial BZT-BCT thin films. High tunability values and excellent temperature and frequency stability enable these epitaxial BZT-BCT thin films as a promising candidate in the application of electrical phase shifter or filters.

## 4. Summary

(110)-oriented epitaxial BZT-BCT thin films on SrRuO_3_-buffered single crystal (110) SrTiO_3_ substrates are prepared by the PLD method. These thin films exhibit diffused phase transition characteristics, reflecting their relaxor-ferroelectric nature. A slim ferroelectric hysteresis loop is observed with a *P_r_* of 2.01 μC/cm^2^ and an *E_c_* of 187 kV/cm. Obvious polarization imprint is observed in their *P*-*E* hysteresis loops. Small leakage currents and high tunability of 70.6% at room temperature are obtained at an electric field of 240 MV/m in these thin-film samples. These epitaxial BZT-BCT thin films exhibit large tunability values ranging from 75.8% to 85.7%, measured at a temperature range from 200 K to 330 K and at a frequency range between 100 Hz and 100 kHz.
